# Diversity Performance Analysis on Multiple HAP Networks

**DOI:** 10.3390/s150715398

**Published:** 2015-06-30

**Authors:** Feihong Dong, Min Li, Xiangwu Gong, Hongjun Li, Fengyue Gao

**Affiliations:** 1 College of Communications Engineering, PLA University of Science and Technology, 88 Houbiaoying Rd., Nanjing 210007, China; E-Mails: xiangwugong@hotmail.com (X.G.); xclhj1985@163.com (H.L.); fengyue80@gmail.com (F.G.); 2 Institute of China Electronic System Engineering Corporation, 13 Dacheng Rd., Beijing 100141, China; E-Mail: limin_sclssw@163.com

**Keywords:** wireless sensor networks, high altitude platform, virtual multiple-input multiple-output, system capacity, shadowed Rician fading, multiple assets in view, average symbol error rate, channel state information

## Abstract

One of the main design challenges in wireless sensor networks (WSNs) is achieving a high-data-rate transmission for individual sensor devices. The high altitude platform (HAP) is an important communication relay platform for WSNs and next-generation wireless networks. Multiple-input multiple-output (MIMO) techniques provide the diversity and multiplexing gain, which can improve the network performance effectively. In this paper, a virtual MIMO (V-MIMO) model is proposed by networking multiple HAPs with the concept of multiple assets in view (MAV). In a shadowed Rician fading channel, the diversity performance is investigated. The probability density function (PDF) and cumulative distribution function (CDF) of the received signal-to-noise ratio (SNR) are derived. In addition, the average symbol error rate (ASER) with BPSK and QPSK is given for the V-MIMO model. The system capacity is studied for both perfect channel state information (CSI) and unknown CSI individually. The ergodic capacity with various SNR and Rician factors for different network configurations is also analyzed. The simulation results validate the effectiveness of the performance analysis. It is shown that the performance of the HAPs network in WSNs can be significantly improved by utilizing the MAV to achieve overlapping coverage, with the help of the V-MIMO techniques.

## 1. Introduction

High altitude platforms (HAPs) are considered to be a novel solution for providing telecommunications services for a wide range of smart wireless sensor network (WSN) devices [1]. In recent years, they have attracted the attention of the telecommunications community [2]. The most important advantages of these networks include their easy and incremental deployment, flexibility, low-cost operation, low propagation delay, high elevation angles, broad coverage, broadcast capability, broadband capability and their ability to be moved around in emergency situations, which are all very suitable for WSNs [3]. Additionally, the advantages of an unconstrained orbital mechanism and low fuel consumption make them the preferred option for a wide range of services, including monitoring, observation, and sensing applications [4].

As an emerging communication platform, the HAP has received growing attention and some progress has been made on this technology. Researchers have proposed consideration of the HAP as a mobile base station in 4G/LTE and 5G networks. It was envisaged that the HAP could improve the coverage while reducing the cost of network construction [5], and the HAP is one of the most promising alternative infrastructures for realizing a wide range of high-data-rate collection applications for WSNs and next-generation wireless networks [6].

The fading of the wireless channel can have a strong negative impact on the link quality of HAP to WSNs devices (named users), so how to effectively deal with the negative impact will be crucial in the HAP network design. Diversity techniques are widely used in wireless communication networks to overcome the negative impact of wireless channel fading. The most common diversity techniques include time diversity, frequency diversity, and space diversity. Compared with time diversity and frequency diversity, space diversity can enhance network performance by configuring multiple antennas at the transmitting and receiving ends to overcome channel fading effects without additional bandwidth [7].

For a specified coverage area, multiple HAPs tend to be used, or a named constellation of interconnected HAPs, to increase the transmission rate, enhance capacity, and improve the quality of service (QoS) in WSNs [8]. A multiple HAP network can achieve a higher data rate by combining the capabilities of several HAPs that individually operate at modest data rate. The responsibilities of each HAP reduce linearly with the number of HAPs in the network [9]. Each HAP can allocate more of its resources to each user, satisfying higher data rate requirements. Therefore, increasing the number of HAPs in the coverage area yields linear increases in the achievable rate of information flow from each user. The impact of component failures, blockage, or rain/cloud cover can be reduced by space diversity if there are redundant communication links [10]. The link redundancy can be provided by overlapping coverage of the HAPs. From the users' perspective, this is also known as multiple assets in view. For example, a mobile sensor device can select, from all of those in view, the HAP with the clearest line of sight, which can reduce service outages and improve availability [11].

Equivalently, at a specified rate, the level of integrity increases with the number of HAPs. The energy per symbol *γ_s_* increases when there are more HAPs in view, as a result of the increased dwell time allowed by the receiving terminal [12]. Then, linear increases in the dwell time result in linear increases in the energy per symbol to noise density ratio (*γ_s_*/*N*_0_) [13]. The integrity will then improve almost exponentially, since the error probability can be approximated as an exponential function of *γ_s_*/*N*_0_. Furthermore, increasing the HAPs in view decreases the probability that users are unable to communicate with a HAP in the network via a direct link due to line-of-sight obstruction by buildings and other tall objects. Consequentially, the multiple viewing angles provided by multiple HAPs improve the availability and rate of the HAP-based network. If properly designed, additional HAPs can also improve the total system capacity of the network [14].

Multiple-input multiple-output (MIMO) techniques, which make use of the spatial dimension by utilizing multiple antennas at the transmitter and receiver, have been proved to be efficient solutions for providing higher data throughput and/or link reliability in WSNs. Due to the very close distance between the antennas in a single HAP, the use of traditional MIMO techniques cannot overcome large scale fading. Additionally, distributed antenna techniques can be introduced to a HAP constellation to form a distributed virtual multiple-input single-output (V-MISO) or virtual MIMO (V-MIMO) HAP network, which will further improve diversity performance to overcome obstruction by buildings and other tall objects, and strengthen the spectrum utilization and reliability of the user data transmission [15].

### 1.1. Related Works and Motivation

Many researches have been investigated on the HAPs communications between stratospheric HAPs and terrestrial terminals. These topics mainly include the HAP channel modeling, constellation of multiple HAPs, and V-MIMO HAPs network, which are as follows:

Michailidis *et al.* [16] proposed a 3-D geometry-based single-bounce reference model for the Rician fading channels in the HAP-MIMO network. The proposed model provides guidelines for the network design and performance analysis of HAP-MIMO networks with line-of-sight (LOS) and non-line-of-sight (NLOS) connections at the L and S frequency bands. Aamir *et al.* [17] provided the channel models associated with an integrated HAP, satellite, and terrestrial network and then applied multiple antenna networks to evaluate and investigate the gains that could be achieved by utilizing MIMO techniques in such networks. Falletti *et al.* [18] considered that the channel experienced by HAPs played a key role for the provision of reliable communications services. He proposed a novel channel model and a related channel simulator especially tailored for HAP-based communication systems. All of these three references [16-18] focus on channel modeling which is critical for our follow-up study.

Chen *et al.* [19] investigated a multiple HAP scenario where all HAP's operate on the same channel. Interfere with each other was assessed in terms of the carrier to interference plus noise ratio (CINR) and spectral efficiency. It is shown that with the number of HAPs increasing from 4, 8 to 16, the system capacity increases almost pro-rata. Huang [20] proposed that the uplink capacity of a CDMA system was improved by using multiple HAPs. By dynamically choosing a serving HAPS among multiple ones in the HAPs network, the required transmit power could significantly be lowered in the proposed scheme, and the overall interference in the uplink can thus be reduced, resulting in an increase of the uplink capacity of the system.

Hult *et al.* [21,22] and Mohammed *et al.* [23] addressed the potential gain of using various compact MIMO antenna array configurations in conjunction with HAP diversity techniques for the first time. It is shown that a compact MIMO-cube antenna array is superior to the vector element antenna since it has twice the number of independent channels, which will give a higher capacity. They also investigated the effects of spatial correlation and mutual coupling between the separate antenna elements on the system performance. However, the papers mainly showed the effects of spatial correlation and mutual coupling between the separate antenna elements of the MIMO-Cube array, and the effects of separation angle between HAPs on system capacity. Celcer *et al.* [24] analyzed the performance of transmit diversity based on space-time block coding (STBC), in particular Alamouti and extended Alamouti schemes, using fixed wide-lobe receive antennas. The performance was compared with the reference receive diversity scheme based on best HAP selection that requires highly directional and steerable antennas in a V-MIMO scenario, with a constellation of multiple HAPs. However, only some experimental data is given, theoretical performance analysis is absent. Mohammed *et al.* [5] considered that there was a very low probability that all radio paths would exhibit deep fade. If all received signals are properly combined, the HAP network performance can be significantly improved. Obviously, these ideal assumptions will not work in practice.

Although the problems of V-MIMO in constellations of interconnected HAPs network have been extensively studied, there are still some vital issues that need to be addressed, especially for the situation with diversity receiving from multiple HAPs in WSNs. There are quite a few works in the literature that have addressed the issue of diversity receiving in WSNs. As it is crucially important for the theoretical research and application of multiple HAP networks, we will provide a theoretical analysis of diversity receiving utilizing V-MIMO techniques in the multiple HAP networks within this paper. The simulation will further prove that the proposed model can improve the quality of communication and reduce the fading margin.

### 1.2. Contributions

The main contributions of this work compared with the previous work are as follows:

- A V-MIMO model is proposed by networking constellation of interconnected HAPs with the concept of MAV. We explain the concept in the users' perspective, and conclude that the improvement in signal-to-noise ratio (SNR) of MAV compared with a single HAP in view is the number of HAP in view.
- The diversity performance is investigated in a shadowed Rician fading, which consists of probability density function (PDF) and cumulative distribution function (CDF) of the received SNR, average symbol error rate (ASER) with BPSK and QPSK.
- The system capacity is analyzed for both perfect channel state information (CSI) and unknown CSI individually. The ergodic capacity with various SNR and Rician factor for different network configurations are investigated.

### 1.3. Organization

The remainder of the paper is organized as follows. Section 2 gives the system model, including the V-MISO and V-MIMO, multiple assets in view, and channel modeling. Section 3 analyzes the path diversity, system outage probability, and investigates the ASER and the system capacity of the multiple HAP network. Section 4 shows the numerical simulations and results. Finally, Section 5 summarizes our conclusions and provides next steps to this work. Table 1 lists the primary notations used in the paper.

## 2. System Model

### 2.1. V-MISO and V-MIMO

**Figure 1:**
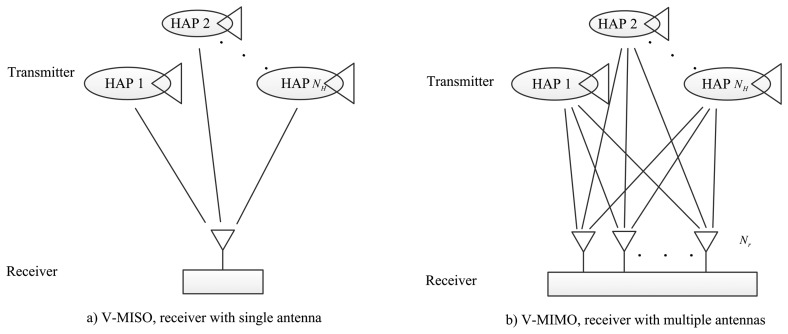
System model.

Assuming that each HAP has a single antenna, a user link is established between the HAP and the sensor device. The HAPs have inter platforms links (IPLs) with each other. There are backhaul links from the HAPs to the ground stations or the satellite backbone network. With the help of synchronous processing at the ground station, each HAP can obtain perfect timing and carrier synchronization. The multiple HAPs can then transmit signals to the receiver at the same frequency in the same time slot. As a result, the multiple HAPs comprise a virtual antenna array (VAA). The *N_H_* HAPs are equivalent to a distributed antenna system with *N_H_* antennas. Each HAP antenna can be considered to be a small antenna on the virtual platform, which consists of multiple platforms. The V-MISO and V-MIMO system models are then established by considering single antenna users and multiple antenna users respectively, as shown in Figure 1. In a true/co-located MIMO HAP network architecture, the multiple antennas are connected to a single transmitter/receiver node, e.g., [17,23]. Due to the close distance between the antennas, it is difficult to solve the large scale shadow fading problem caused by blocking from large obstacles. However, in the V-MIMO HAP network, each antenna is far apart from each other, so an independent large-scale fading channel is available, and a better diversity gain is obtained to prevent shadow fading.

### 2.2. Multiple Assets in View

If the sensor device can establish links with multiple HAPs, it is defined as multiple assets in view (MAV) or multiple HAPs in view. The MAV can provide a lot of extra benefits from the users' perspective: Rate and integrity improvements, signal routing flexibility, cooperative relay, and overcoming instability of platform that degrades system performance, *etc*.

It is known that the SNR of receiver is a identical to a filled aperture of the same physical collecting area in the antenna. If there are *N_H_* assets in view, as depicted in Figure 1a, which each have a transmitter antenna aperture diameter of *D_H_*, then the receiver antenna measures the radiation field and the signals from each of the different antennas, and combines this to deliver a single waveform to the detector. In the most general case, the input signal power varies across the array. Assuming unit impedance throughout, the average signal power is given by [9]:

(1) $$\text{E}\left[S_{i}\right]=\text{E}\left[\frac{1}{N_{H}}\sum_{j=1}^{N_{H}}s_{ij}^{2}\right]$$

where E [·] denotes the expectation operator and *s_ij_* are the proportional to the envelope of the RF signal voltage for the *j*th transmitter antenna.

Assuming the signal strength across the antenna is constant, *s_ij_* = *s_ik_* = *s_i_*, and the signal power per transmitter antenna is $S_{i}=s_{i}^{2}$. If all signals are co-phased by a bank of phase shifters, the output signal voltage after integration is *N_H_s_i_* and the output signal power of the receiver can be represented as $S_{0}=N_{H}^{2}S_{i}$. The noise from each antenna is an independent and identically distributed (i.i.d.) complex Gaussian random variable, uncorrelated with the signals, each following a ${ℵ}_{C}\left(0,\sigma_{r}^{2}\right)$ distribution. The average noise power is:

(2) $$\text{E}\left[N_{i}\right]=\text{E}\left[\frac{1}{N_{H}}\sum_{j=1}^{N_{H}}n_{ij}^{2}\right]$$

The square of the noise voltage is the receiving noise power:

(3) $$N_{0}={\left(n_{0}\right)}^{2}={\left(\sum_{j=1}^{N_{H}}n_{ij}\right)}^{2}=\sum_{j=1}^{N_{H}}n_{ij}^{2}+\sum_{j=1}^{N_{H}}\sum_{k=1}^{N_{H}}n_{ij}n_{ik}$$

where *n_ij_* denote the noise between the *i*th receiving antenna and the *j*th transmitter antenna.

Since the noise is uncorrelated, it does not add up in phase and so $\overset{N_{H}}{\underset{j=1}{\text{∑}}}\overset{N_{H}}{\underset{k=1}{\text{∑}}}n_{ij}n_{ik}=0$ and E[*N_i_*] = *N_H_*E [*N*_0_]. The output SNR is therefore equal to:

(4) $$\gamma_{0}=\frac{S_{0}}{\text{E}\left[N_{0}\right]}=\frac{N_{H}S_{i}}{\text{E}\left[N_{i}\right]}=N_{H}\gamma_{1}$$

where *γ*_1_ denotes the SNR of a single HAP in view. If the QPSK is used and the required BER is 10^−6^, the corresponding threshold of SNR for the sensor device is 5.3 dB. Assuming the value of *γ*_1_ provided by a single HAP is 1.6 dB, and there are four HAPs and a feasible data transmission can be achieved. However, it will be impossible when there is only one HAP.

The improvement in SNR of MAV compared with a single asset in view is *N_H_*, the number of assets in view, therefore contributing to the rate and integrity improvements.

### 2.3. Channel Model

During propagation, the wireless signal is normally influenced by path loss, shadows, and multipath fading. The path loss is the mean of the wireless signal transmission attenuation in large scale fading (usually across tens to hundreds of wavelengths). In the HAPs communication scenario, it is generally believed that the Rician fading characteristic is appropriate, which includes the LOS and NLOS components [12]. The LOS component is modeled as a shadowed free space propagation loss. If α^2^ and 2σ^2^ denote the power of the LOS component and NLOS component respectively, the normal PDF of the signal can be represented as given in [25] by:

(5) $$\begin{array}{cc} f\left(s|\alpha ,\sigma \right)=\frac{s}{\sigma^{2}}\exp\left\{-\frac{s^{2}+\alpha^{2}}{2\sigma^{2}}\right\}K_{0}\left(\frac{s\alpha }{\sigma^{2}}\right), & s\geq 0 \end{array}$$

where *K*_0_(·) denotes the zero order modified Bessel function of the first kind.

However, the LOS component is frequently shadowed by trees, buildings, and other obstacles, therefore if we further take the shadows into consideration, the LOS component in Equation (5) obeys a lognormal distribution, and its distribution function is:

(6) $$f_{L_{s}}\left(l\right)=\frac{1}{\sqrt{2\pi }\delta l}\exp\left(-\frac{{\left(\ln l-\ln L_{0}\right)}^{2}}{2\delta^{2}}\right)$$

where *L_s_* denotes the shadowed LOS, *δ* is the severity of the shadow fading, typically in the range 1.5–7 dB and *L*_0_ denotes the average path loss. A larger value of *δ* means more serious shadows in the LOS signal. In the proposed model, a typical value of *L*_0_ is 129.1 dB when the HAPs are at an altitude of 22 km.

For mathematical analysis convenience, Equation (6) can be considered to be equivalent to the Gamma distribution given by [26]:

(7) $$f_{L_{s}}\left(l\right)=\frac{l^{m_{s}-1}}{\Gamma \left(m_{s}\right)\left(\Omega_{s}/m_{s}\right)}\exp\left(-l/\left(\Omega_{s}/m_{s}\right)\right)$$

where Γ(*m_s_*) is the Gamma function, *m_s_* = 1/ exp(*δ*^2^ − 1), and Ω*_s_* = *L*_0_ exp(*δ*^2^/2).

The NLOS component refers to the rapid fluctuation of the received signal amplitude based on the shadow slow fading. Generally, the Nakagami distribution is used, and its distribution function is given by:

(8) $$f_{L_{m}}\left(l\right)=\frac{1}{\Gamma \left(m\right)}{\left(\frac{m}{{\bar{L}}_{s}}\right)}^{m}l^{m-1}\exp\left(-\frac{m}{L_{s}}l\right)$$

where *L_m_* denotes the loss of multipath fading (NLOS) and *m* ≥ 0 is the fading factor. Typically *L_m_* is in the rang 7–12 dB and a larger *L_m_* means more serious NLOS signal loss. A larger *m* means better channel conditions, and it is typically in the rang 1.2–10 dB. *L̄_s_* = E(*r*^2^) represents the average power of multipath fading, which obeys the Gamma distribution.

Therefore, the comprehensive channel fading model *L_f_* can be represented as given in [25] by:

(9) $$f_{L_{f}}\left(l\right)=\int_{0}^{\infty }f_{L_{m}}\left(l|x\right)f_{L_{s}}\left(x|L_{0}\right)dx=\frac{2}{\Gamma \left(m\right)\Gamma \left(m_{s}\right)}{\left(\frac{mm_{s}}{{\left(4\pi d/\lambda \right)}^{2}}\right)}^{\frac{m+m_{s}}{2}}l^{\frac{m+m_{s}-2}{2}}K_{m_{s}-m}\left(2\sqrt{\frac{mm_{s}}{{\left(4\pi d/\lambda \right)}^{2}}l}\right)$$

where *K_m_s__*_−_*_m_*(·) denotes the *m_s_* − *m* order modified Bessel function of the second kind.

Due to the far distance between HAPs, we assume that each sub-channel obeys the same distribution given in Equation (9). Therefore, the channel model for the multiple HAP V-MIMO network is given by:

(10) $$\text{H}\left(f\right)=\sqrt{\frac{\kappa }{\kappa +1}}{\text{H}}_{\text{LOS}}\left(f\right)+\sqrt{\frac{1}{\kappa +1}}{\text{H}}_{\text{NLOS}}\left(f\right)$$

where *κ* = α^2^/(2σ^2^) is the Rician factor, **H***_los_* (*f*) represents the channel matrix of the shadowed LOS path, and **H***_NLOS_* (*f*) denotes the scattering and NLOS path. Note that the channel model is remarkably different from that in [16–18], in which the system model is a true MIMO-HAP architecture.

In addition, when the signal bandwidth B is much lower than the carrier frequency *f_c_*, the channel will be a frequency flat-fading channel. At this point, the channel model can be further simplified as:

(11) $$\text{H}=\sqrt{\frac{\kappa }{\kappa +1}}{\text{H}}_{\text{LOS}}+\sqrt{\frac{1}{\kappa +1}}{\text{H}}_{\text{NLOS}}$$

## 3. Performance Analysis

In this paper, the authors are asserting that the network performance can be significantly improved by utilizing MAV to achieve overlapping coverage. The essential benefit of MAV is the path diversity. Multiple HAPs provide more nodes in the network for information flow than a single HAP. Therefore, path diversity can definitely improve the availability of HAPs networks.

### 3.1. Path Diversity Analysis

Path diversity makes use of two or more statistically independent radio paths to improve the transmission reliability [7]. The probability that all radio paths exhibit deep fading is very low, so if all received signals are properly combined, the network performance can be significantly improved. The statistically independent paths can be obtained by [27]: Spatial diversity, frequency diversity, temporal diversity, angle diversity, and polarization diversity.

Clearly, in order to increase the transmission reliability, the diversity techniques reduce the capacity of the communication network by sacrificing radio resources. In the case of diversity reception, the best received signal should be selected (selection diversity) or all received signals should be combined into one signal for data estimation (combination diversity). Due to the predominant LOS channel conditions in a HAP operating environment, propagation channels are highly correlated and most diversity techniques are not applicable. The exception may be spatial diversity on the ground or use of multiple HAPs [11].

The diversity gain here is that the multiple antennas in the system utilize the non-correlation of the received signal in each antenna, reduce the amplitude of fading by combining signals, and then achieve the performance gain. The principle is that when each channel is affected by independent fading, the HAPs transmit multiple copies of the same signal in different channels, which can significantly reduce probability of the deep fading in all copies at the same time. Thus, the wireless channel fading can be overcome and the transmission performance is also improved. Additionally, different incoherent signals paths are diversely processed by the receiver, which can greatly reduce the bit error rate (BER) of the information, and effectively resist blocking interference.

We assume that the number of HAPs is *N_H_*, the number of receiving antennas for the sensor device is *N_r_*, and the transmitting signal vector is **s** (*t*) ∈ ℂ*^N_H_^*^×1^ which has a power expectation of E [|**s**(*t*)|^2^] = 1. The beamforming vector in the V-MIMO transmitter is **w** (*N_H_* × 1). The signal is transmitted through a channel gains matrix of complex fading **H**(*t*) = [*h_ij_*(*t*)]*_N_r__*_×_*_N_H__* ∈ ℂ*^N_r_^*^×^*^N_H_^*, and is received using the *N_r_* × 1 beamforming vector **v** in the receiver. Ultimately, the received signals **y** (*t*) ∈ *C^N_r_^*^×1^ can then be represented as:

(12) $$\text{y}\left(t\right)={\text{v}}^{H}\left[\text{Hw}\sqrt{P_{\text{s}}}\text{s}\left(t\right)+\text{n}\left(t\right)\right]={\text{v}}^{H}\text{Hw}\sqrt{P_{s}}\text{s}\left(t\right)+{\text{v}}^{H}\text{n}\left(t\right)$$

where the superscript *H* denotes the hermitian conjugate transpose operator and *P_s_* is the transmitted power. The channel matrix **H**(*N_r_* × *N_H_*) is shadowed Rician fading, whose NLOS element [**H***_NLOS_*] *_i,j_* is the i.i.d. complex Gaussian distribution $\sqrt{1/\left(\kappa +1\right)}\times {ℵ}_{C}\left(0,1\right)$, and the amplitude of LOS element [**H***_LOS_*] *_i,j_* is $\sqrt{\kappa /\left(\kappa +1\right)}$, which obeys the lognormal distribution, as given by Equation (7). **n**(*t*) ∈ ℂ*^N_r_^*^×1^ denotes the vector of additive white Gaussian noise (AWGN), whose elements follow the i.i.d. ${ℵ}_{c}\left(0,\sigma_{r}^{2}\right)$ distribution, and E [**n** (*t*) **n***^H^* (*t*)] = *N*_0_**I***_N_r__*. Then, the SNR of receiver is therefore given by:

(13) $$\gamma ={\left|{\text{v}}^{H}\text{Hw}\right|}^{2}\frac{P_{\text{s}}\text{E}\left[{\left|s\left(t\right)\right|}^{2}\right]}{{\text{v}}^{H}\text{E}\left[\text{n}\left(t\right){\text{n}}^{H}\left(t\right)\right]\text{v}}={\left|{\text{v}}^{H}\text{Hw}\right|}^{2}\frac{P_{s}}{{\text{v}}^{H}N_{0}{\text{I}}_{N_{r}}\text{v}}={\left|{\text{v}}^{H}\text{Hw}\right|}^{2}\frac{P_{s}}{N_{0}}={\left|{\text{v}}^{H}\text{Hw}\right|}^{2}\gamma_{s}$$

where *γ_s_* = *P_s_*/*N*_0_ is the SNR of the virtual transmitter.

Eigenvalue decomposition of the cross-correlation matrix should be firstly performed, ahead of solving the beamforming vectors **w** and **v** to maximize the SNR of receiver. It can be represented as:

(14) $${\text{H}}^{\text{H}}H=\left[{\text{u}}_{1},{\text{u}}_{2},\cdots ,{\text{u}}_{N_{H}}\right]\text{diag}\left(\lambda_{1},\lambda_{2},\cdots ,\lambda_{N_{H}}\right){\left[{\text{u}}_{1},{\text{u}}_{2},\cdots ,{\text{u}}_{N_{H}}\right]}^{H}$$

where λ_1_ > λ_2_ > ⋯ λ*_N_* > 0 are the nonzero distinct eigenvalues, and λ_1_ and **u**_1_ denote the maximal eigenvalue and its corresponding characteristic vector respectively. The optimal beamforming vector can be given by:

(15) $$\text{w}={\text{u}}_{1}$$

(16) $$\text{v}={\text{Hu}}_{1}/{\left\|{\text{Hu}}_{1}\right\|}_{F}$$

where ‖·‖*_F_* represents the Frobenius norm operator. Further, the maximum SNR of the receiver is:

(17) $$\gamma =\lambda_{1}\gamma_{s}$$

The beamforming schemes in Equations (15) and (16) are the maximal-ratio combining (MRC) and maximal-ratio transmitting (MRT) respectively. Letting *N_rH_* = min {*N_H_*, *N_r_*}, *N_Hr_* = max {*N_H_*, *N_r_*}, the PDF of λ_1_ can be then represented as:

(18) $$f_{\lambda_{1}}\left(x\right)=\frac{\exp\left(-tr\left(\lambda \right)\right)\left|\Psi \left(x\right)\right|tr\left(\Psi^{-1}\left(x\right)\Psi_{N_{rH}}\left(x\right)\right)}{\left|V\right|{\left(\Gamma \left(N_{Hr}-N_{rH}+1\right)\right)}^{N_{rH}}}U\left(x\right)$$

where λ = *diag* (λ_1_, λ_2_, ⋯, λ*_N_H__*), |·| denotes the determinant operator, *tr*(·) denotes the trace operator, **Ψ**(*x*) is the *N_rH_* × *N_rH_* matrix function, with entries ${\left\{\Psi \left(x\right)\right\}}_{i,j}=\int_{0}^{x}z^{t-1}\exp{\left(-z_{0}\right)}_{0}F_{1}\left(N_{Hr}-Nr_{H}+1;\lambda_{j}z\right)dz,i,j=1,2,\cdots ,N_{rH}$. *_p_F_q_*(⋅;⋅.) is the generalized hypergeometric function with parameters *p* and *q*. Γ (·) is the gamma function. $\left|V\right|=\overset{N_{rH}}{\underset{i<j}{\text{∏}}}\left(\lambda_{i}-\lambda_{j}\right)$. *U*(*x*) is the unit step function. **Ψ***N_rH_*(*x*) is the *N_rH_* × *N_rH_* matrix, with entries ${\left\{\Psi_{N_{rH}}\left(x\right)\right\}}_{i,j}=x_{N_{rH}}^{N_{Hr}-i}\exp{\left(-x\right)}_{0}F_{1}\left(N_{Hr}-N_{rH}+1;\lambda_{j}x\right),i,j=1,2,\cdots ,N_{rH}$.

Using Equation (18), the PDF of the receiving SNR *γ* in Equation (13) can be obtained to be:

(19) $$f_{\gamma }\left(x\right)=\frac{1}{\gamma_{s}}f_{\lambda_{1}}\left(x/\gamma_{s}\right)=\frac{\exp\left(-tr\left(\lambda \right)\right)\left|\Psi \left(x\sigma^{2}/\delta^{2}\gamma_{s}\right)\right|tr\left(\Psi^{-1}\left(x\sigma^{2}/\delta^{2}\gamma_{s}\right)\Psi_{rH}\left(x\sigma^{2}/\delta^{2}\gamma_{s}\right)\right)}{P_{s}\delta^{2}/\sigma^{2}\left|\text{V}\right|{\left(\Gamma \left(N_{Hr}-N_{rH}+1\right)\right)}^{N_{rH}}}U\left(x\right)$$

Therefore, the CDF of the receiving SNR *γ* can be written as:

(20) $$F_{\gamma }\left(x\right)=\int_{0}^{x}f_{\gamma }\left(t\right)dt=\frac{\exp\left(-tr\left(\lambda \right)\right)|\Psi \left(x\sigma^{2}/\delta^{2}\gamma_{s}\right)}{\left|\text{V}\right|{\left(\Gamma \left(N_{Hr}-N_{rH}+1\right)\right)}^{N_{rH}}}$$

There are two special cases that can simplify the process above. In the first case, if the Rician factor is large enough, *i.e.*, the LOS component of **H** is stronger and the signal is only slightly shadowed, then the NLOS component can be ignored. Then the matrix **H** has only a single nonzero eigenvalue and the PDF of λ_1_ can be reduced to:

(21) $$f_{\lambda_{1}}\left(x\right)=\frac{\exp\left(-\lambda_{1}\right)\left|\Psi \left(x\right)\right|tr\left(\Psi^{-1}\left(x\right)\Psi \left(x\right)\right)}{\Gamma \left(N_{Hr}-N_{rH}+1\right)\lambda_{1}^{N_{rH}-1}\prod_{k=1}^{N_{rH}-1}\Gamma \left(N_{Hr}-k\right)\Gamma \left(N_{rH}-k\right)}U\left(x\right)$$

In the second case, if the Rician factor is small enough, *i.e.*, the NLOS component of **H** is stronger and the signal is deeply shadowed, then the LOS component can be ignored. Then the **H** matrix has up to *N_rH_* nonzero eigenvalues. The PDF of λ_1_ can be then further represented as:

(22) $$f_{\lambda_{1}}\left(x\right)=\sum_{i=1}^{N_{rH}}\sum_{m=\left|N_{r}-N_{H}\right|}^{\left(N_{H}+N_{r}\right)i-2i^{2}}d_{i,m}\frac{i^{m+1}}{m!}x^{m}\exp\left(-ix\right)U\left(x\right)$$

where *d_i_*_,_*_m_* is given by Dighe ([28], Equation (24)):

(23) $$d_{i,m}=\frac{\Gamma \left(i+1\right)C_{i,m}}{m^{i+1}\left(\overset{N_{rH}}{\underset{l=1}{\text{∏}}}\Gamma \left(N_{r}-l+1\right)\Gamma \left(N_{H}-l+1\right)\right)}$$

where *C_i_*_,_*_m_* denotes the coefficient of its corresponding *x^m^* exp (−*ix*) item, used for expansion of the derivative of the determinant for the matrix **S**(*x*). **S**(*x*) is the *N_r_* × *N_r_* Hankel matrix [28] and it can be described by *S*(*x*)*_k_*_,_*_l_* = Γ(*N* − *L* + *k* + *l* − 1, *x*), where Γ (·, ·.) is the incomplete gamma function and with expression $\Gamma \left(k,x\right)=\int_{0}^{x}t^{k-1}\exp\left(-t\right)dt=\left(k-1\right)!\left[1-\exp\left(-x\right)\overset{k-1}{\underset{m=0}{\text{∑}}}\frac{x^{m}}{m!}\right]$.

In the first case, since **H** will degenerate into a simple matrix with only a single nonzero eigenvalue, *i.e.*, the rank of **H** is 1, it can be analyzed relatively easily. Thus, we only provide results for the second case, where there are more obvious multipath components used to achieve a higher diversity performance in the HAP network. Furthermore, by using Equation (22), the PDF and CDF of receiving SNR *γ* in Equation (13) can be written as:

(24) $$f_{\gamma }\left(x\right)=\sum_{i=1}^{N_{rH}}\sum_{m=\left|N_{H}-N_{r}\right|}^{\left(N_{H}+N_{r}\right)i-2i^{2}}d_{i,m}{\left(\frac{i}{\gamma_{s}}\right)}^{m+1}\frac{x^{m}}{m!}\exp\left(-\frac{i}{\gamma_{s}}x\right)$$

(25) $$F_{\gamma }\left(x\right)=1-\sum_{i=1}^{N_{rH}}\sum_{m=\left|N_{H}-N_{r}\right|}^{\left(N_{H}+N_{r}\right)i-2i^{2}}d_{i,m}\exp\left(-\frac{i}{\gamma_{s}}x\right)\left(\sum_{t=0}^{m}\frac{i^{t}}{t!}{\left(\frac{x}{\gamma_{s}}\right)}^{t}\right)$$

### 3.2. Outage Probability

The outage probability is an important statistical measurement to assess the quality of service provided by the wireless communication network. Typically, the outage probability should be in the range 10^−5^ – 10^−6^, and a smaller outage probability value denotes better diversity performance. It can be defined as the probability that the end-to-end instantaneous SNR is lower than a specified SNR threshold value *γ*_th_ which is required for satisfactory reception. By replacing *x* with *γ_th_* in Equation (20), the outage probability can be calculated by [29]:

(26) $$P_{\text{out}}=\mathrm{Pr}\left(\gamma \leq \gamma_{th}\right)=F_{\gamma }\left(\gamma_{th}\right)=\frac{\exp\left(-tr\left(\lambda \right)\right)|\Psi \left(x\sigma^{2}/\delta^{2}\gamma_{th}\right)}{\left|\text{V}\right|{\left(\Gamma \left(N_{Hr}-N_{rH}+1\right)\right)}^{N_{rH}}}$$

We can observe that *P_out_* is a function of *N_h_*, *N_r_*, *γ*, and *γ_th_*. Their significance on the diversity performance based on multiple HAP networks will be evaluated in Section 4.

### 3.3. Average Symbol Error Rate

There are two main methods to get the ASER: A moment generating function or CDF. In this paper, the end-to-end CDF of the receiving SNR has already been obtained using Equation (20). Therefore, we prefer to use the CDF method to calculate the ASER. The typical value of necessary ASER is 10^−6^, and the smaller value of ASER means the better diversity performance. Based on Ref. [30], and the modulating M-PSK and M-PAM using the Gray code mapping constellation, the ASER can be represented or approximated as $P_{e}=\text{E}\left[aQ_{1}\left(\sqrt{b\gamma }\right)\right]$, where *Q*_1_ (·) denotes the Gaussian function defined as $Q_{1}\left(x\right)=\left(1/\sqrt{2\pi }\right)\int_{x}^{\infty }\exp\left(-t^{2}/2\right)dt$. The letters *a* and *b* are constants related to the modulation method. For example, when *a* = 1, *b* = 1, the corresponding modulation method is BPSK. *a* = 2, *b* = sin^2^(*π*/*M*) corresponds to M-PSK modulation, while *M* ≥ 4, *a* = 2 (*M* − 1)/*M*, *b* = 3/(*M*^2^ − 1) corresponds to M-PAM modulation. To obtain an expression for *P_e_*, we can integrate the PDF of *γ*. After simple variable substitution, *P_e_* can be reformulated as:

(27) $$P_{e}=\frac{a}{2}\sqrt{\frac{b}{\pi }}\int_{0}^{\infty }\frac{\exp\left(-bx\right)}{\sqrt{x}}F_{\gamma }\left(x\right)dx$$

Substituting Equation (20) into Equation (27)*P_e_* can be written as:

(28) $$P_{e}=\frac{a}{2}\sqrt{\frac{b}{\pi }}\int_{0}^{\infty }\frac{\exp\left(-bx\right)}{\sqrt{x}}\frac{\exp\left(-tr\left(\lambda \right)\right)|\Psi \left(x\sigma^{2}/\delta^{2}\gamma_{s}\right)}{\left|\text{V}\right|{\left(\Gamma \left(N_{Hr}-N_{rH}+1\right)\right)}^{N_{rH}}}dx$$

### 3.4. System Capacity

The system capacity is an important indicator to measure the effectiveness of the wireless communication network. Unlike the AWGN channel, due to the random characteristic of the wireless fading channel, there is no uniform definition of system capacity applicable to all fading situations. In a wireless fading environment, indicators that evaluate the channel capacity include ergodic capacity and outage capacity.

The ergodic capacity *C_erg_* reflects the average transmission rate of the channel, is mainly used for communication services that are insensitive to delay, e.g., non-real-time multi-media data in multi-media WSNs. The outage capacity is defined as the average transmission rate that channels can provide under the condition of a specified outage probability value *ε*. In this paper, we will mainly investigate the ergodic capacity. We assume that the adaptive transmission scheme is adopted, *i.e.*, each sub-channel dynamically transmits signal at the optimal data rate and power, based on the characteristics of the channel at that point in time. Various data bits are allocated to each sub-channel during each transmission period, and power allocation obeys the water-filling theorem. The principle of this algorithm is that the HAPs adaptively allocate transmitted power according to known or perfect CSI based on a predetermined guideline. Generally, more power is allocated to better sub-channels and less power is allocated to poorer sub-channels. Therefore, the ergodic capacity is the expectation of the total capacity of all sub-channels, which is given by:

(29) $$C_{\text{erg}}=\text{E}\left(\sum_{i=1}^{r}\log_{2}\left(1+\frac{P_{i}}{N_{0}}\lambda_{i}^{2}\right)\right)$$

where *r* ≤ min(*N_H_*, *N_r_*) is the rank of the transmitting channel, *i.e.*, the conversion of the channel matrix of the multiple HAP V-MIMO network to r mutually independent parallel sub-channels. *P_i_* denote the allocated power to the *i*th sub-channel and $P_{s}=\overset{r}{\underset{i=1}{\text{∑}}}P_{i}$. The optimal power can be given by $P_{i}^{\text{opt}}=\max\left\{\left(\mu -N_{0}/\lambda_{i}^{2}\right),0\right\}$ according to the water-filling algorithm, where λ*_i_* is the gain of the *i*th sub-channel.

However, the CSI may be unknown to HAPs in general, so the average energy allocation scheme will then be used, *i.e.*, each sub-channel has equal power allocation *P_i_* = *P_s_*/*N_H_* [31]. By using the Shannon channel capacity formula, the capacity in our constellation of HAPs network can be written as:

(30) $$C_{\text{erg}}=\text{E}\left(W\sum_{i=1}^{r}\log_{2}\left(1+\frac{\lambda_{i}P_{s}}{N_{H}\sigma^{2}}\right)\right)=\text{E}\left(W\log_{2}\prod_{i=1}^{r}\left(1+\frac{\lambda_{i}P_{s}}{N_{H}\sigma^{2}}\right)\right)=\text{E}\left(W\log_{2}\left|I_{N_{rH}}+\frac{P_{s}}{N_{H}\sigma^{2}}Q\right|\right)$$

where *W* denotes the bandwidth of the sub-channel, and **Q** is the Wishart matrix, defined as:

(31) $$\text{Q}=\{\begin{array}{c} {\text{HH}}^{\text{H}},N_{\text{H}}>N_{r}\\ H^{H}H,N_{H}\leq N_{r} \end{array}$$

## 4. Results and Discussion

The numerical results are provided in this section, which illustrate our previously stated analytical expressions for the diversity performance of the HAP constellations over V-MIMO shadowed Rician fading channels. The simulations were carried out using the MATLAB R2014a simulator. In order to implement the multiple HAP networks model, a subfunction of channel fading matrix was used with LOS and NLOS components. The LOS component follows the the lognormal distribution, and the NLOS component follows the i.i.d. complex Gaussian distribution $\sqrt{1/\left(\kappa +1\right)}\times {ℵ}_{C}\left(0,1\right)$. The HAPs and the sensor device are described in the main function. The PDF and CDF of the received SNR is simulated in the first step. The outage probability, ASER for BPSK and QPSK, and capacity are carried for various configurations in order to make effective comparisons. Table 2 lists the parameters used in simulations and their values.

**Figure 2:**
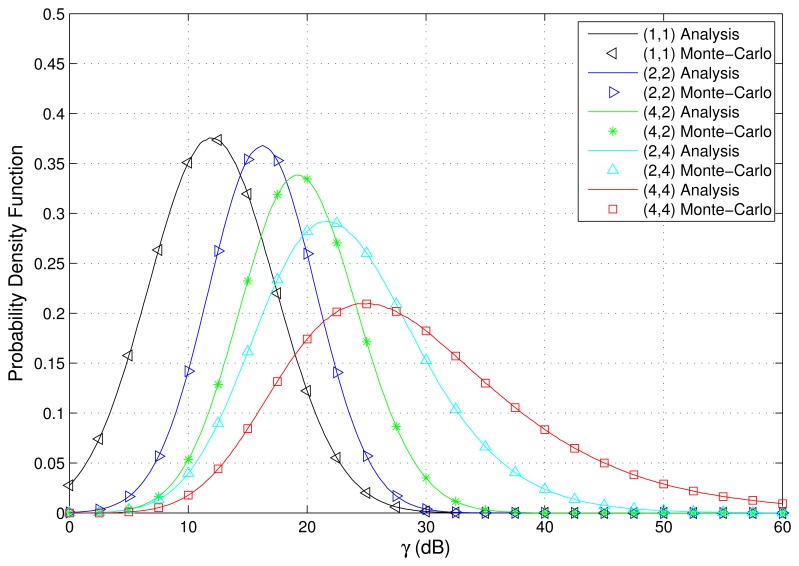
PDF of received SNR with various HAP and user antenna configurations (*κ* = 1).

**Figure 3:**
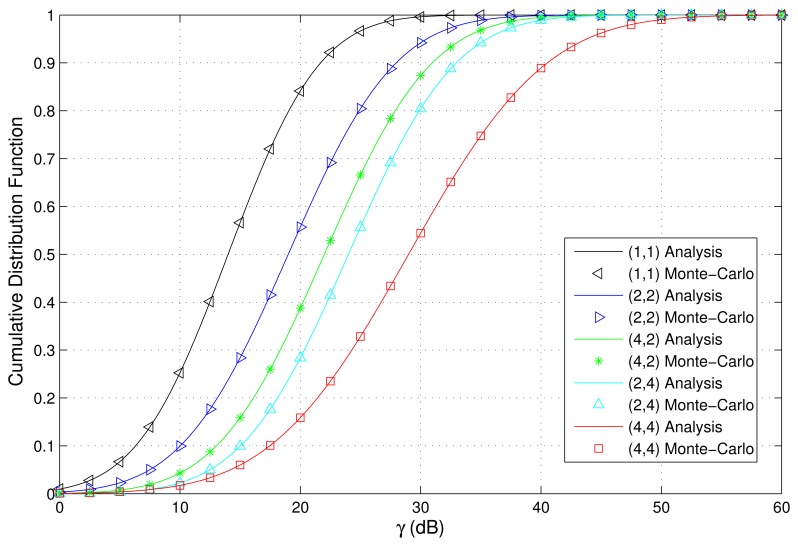
CDF of received SNR with various HAP and user antenna configurations (*κ* = 1).

**Figure 4:**
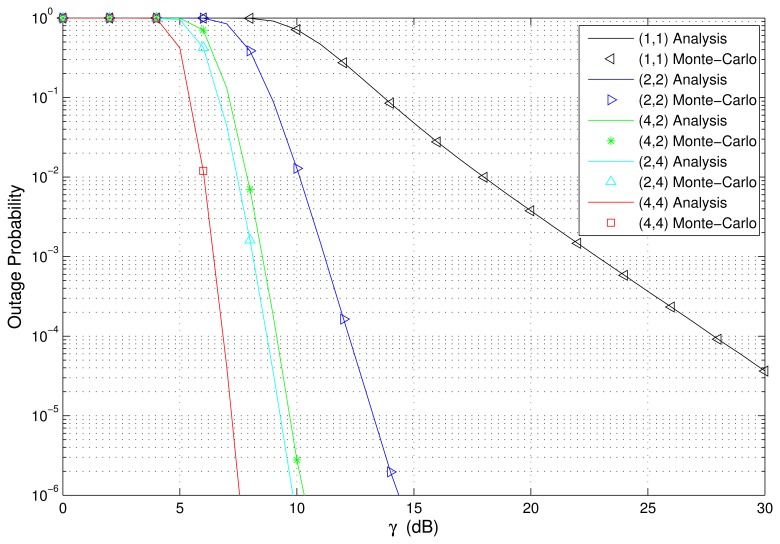
Outage probability versus the average SNR with various HAP and user antenna configurations (*γ_th_* = 10 dB).

**Figure 5:**
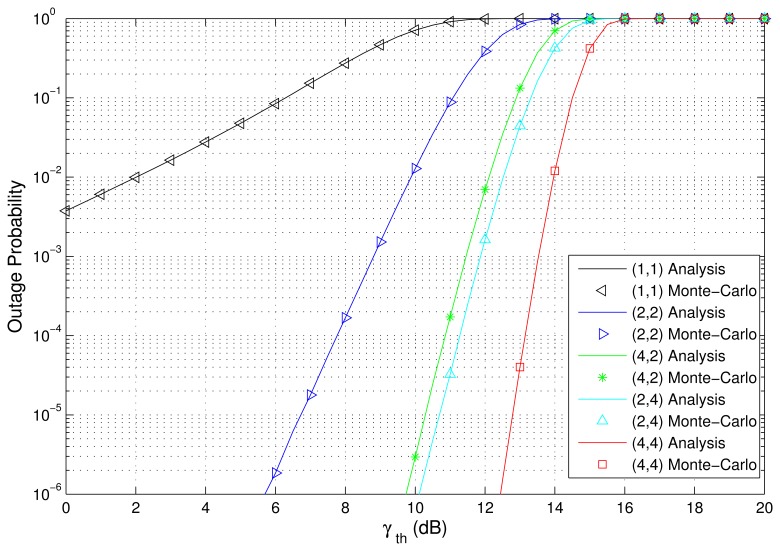
Outage probability versus the SNR threshold *γ_th_* with various HAP and user antenna configurations (*γ* = 10 dB).

**Figure 6:**
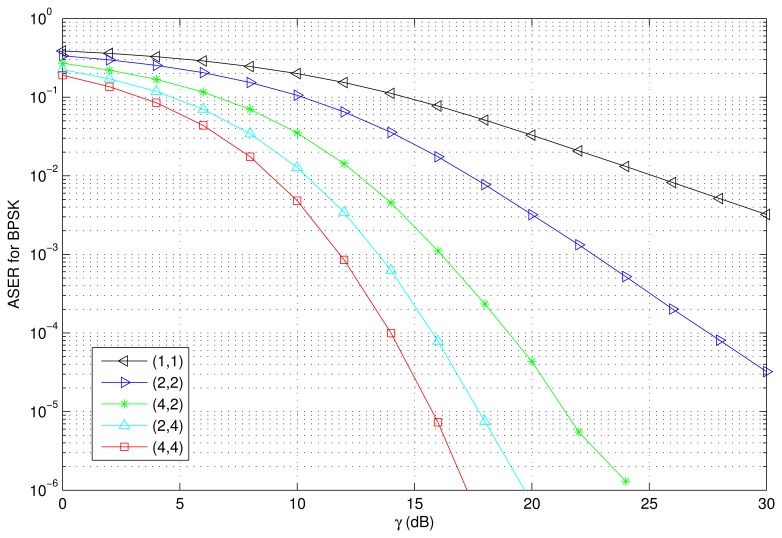
ASER versus the average SNR with various HAP and user antenna configurations (BPSK, *i.e.*, *a* = 1,*b* = 1; *κ* = 1).

**Figure 7:**
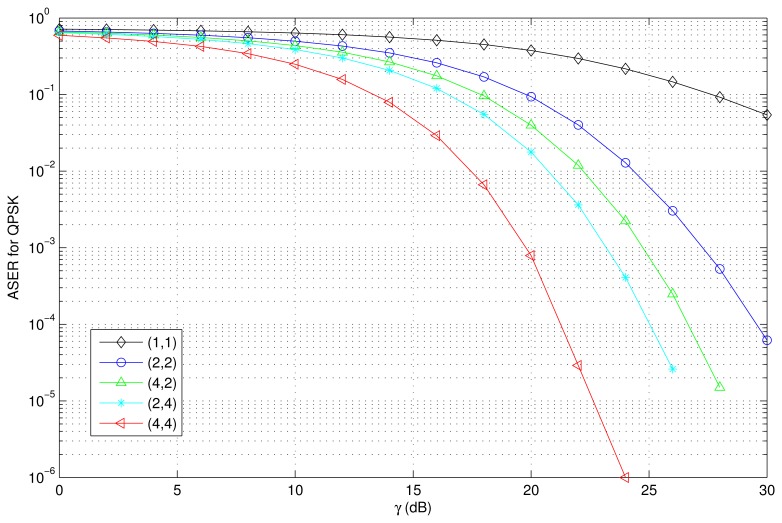
ASER versus the average SNR with various HAP and user antenna configurations (QPSK, *i.e.*, *a* = 2,*b* = sin^2^ (*π*/4); *κ* = 1).

**Figure 8:**
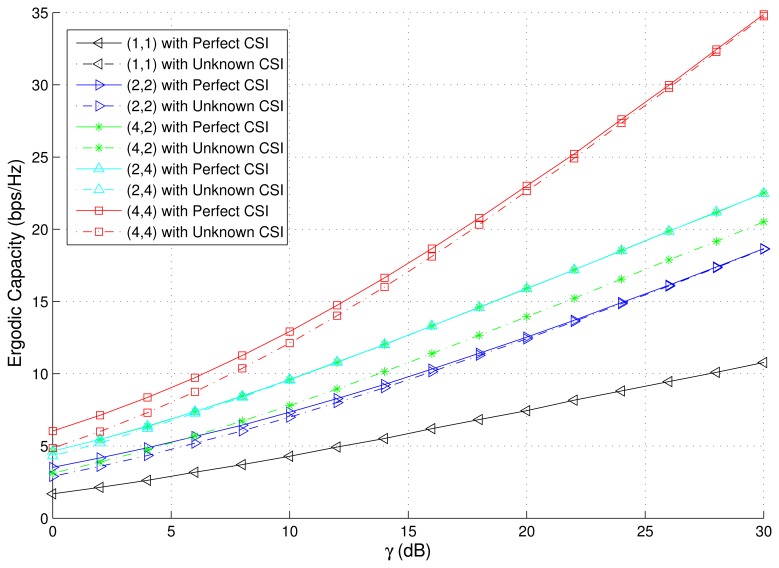
Ergodic capacity changes along with variation of SNR for different network configurations (perfect CSI and unknown CSI).

**Figure 9:**
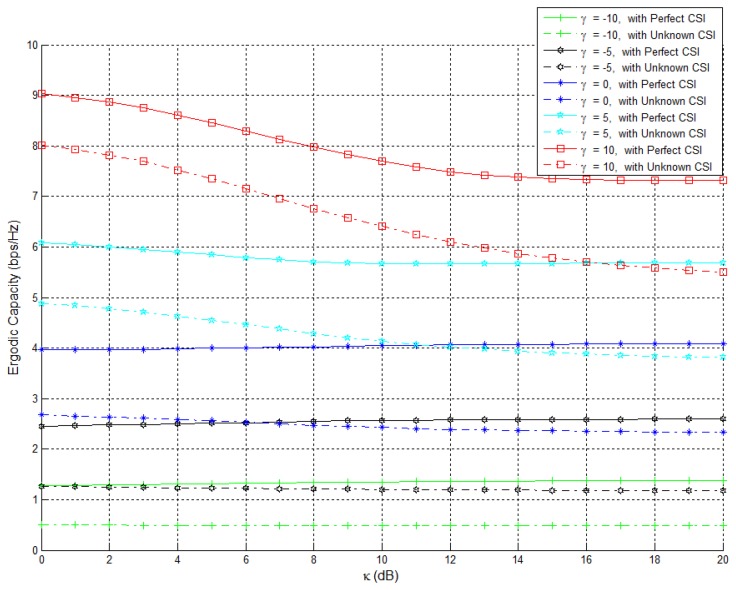
Ergodic capacity changes along with Rician factor under different SNR (*N_H_* = *N_r_* = 4).

The PDF and CDF of the received SNR under different HAPs and user antenna configurations are shown in Figures 2 and 3, respectively. Figures 4 and 5 depict the outage probability versus the average SNR *γ* and SNR threshold *γ_th_*. The ASER for BPSK and QPSK is given in Figures 6 and 7 respectively. The ergodic capacity with various SNR under different HAP and user antenna configurations is shown in Figure 8, for both perfect CSI and unknown CSI. Finally, the ergodic capacity with various Rician factors *κ* is depicted in Figure 9 under different SNR conditions. A comprehensive analysis of the diversity reception performance in multiple HAP networks is described. In all the figures below, the label (*N_H_*, *N_r_*) denotes the number of HAPs and the number of receiver antennas. Since it is assumed that each HAP installs one downlink antenna, the number of transmitter antennas is equal to the number of HAPs *N_H_*. Figures 2, 3, 4 and 5 depict the results of analysis and Monte Carlo, respectively. We run the simulated program 10^4^ times and take the average of results to observe whether the Monte Carlo results are consistent with the performance analysis. In each time, the channel matrix is generated randomly with the same parameters.

Firstly, the influence of the HAP and user antenna configurations on the PDF and CDF of the received SNR is investigated. Assuming *N_H_* is 1,2,4, *N_r_* is 1,2,4, and five representative network configurations are considered, which are (1,1), (2,2), (4,2), (2,4), and (4,4). When *κ* = 1, Figures 2 and 3 depict the PDF and CDF of the received SNR. We can observe that the results of Monte Carlo are completely consistent with that of the previous performance analysis, thus further proving the accuracy and effectiveness of the approach proposed in this paper. Figures 2 and 3 indicate that increasing the number of HAPs and antenna elements improves the opportunity for the user to achieve larger SNR values, implying a better performance.

Secondly, Figures 4 and 5 show the outage probability versus the average SNR and SNR threshold *γ_th_*. We can observe that the performance of (2,4) is better than that of (2,2). This is because even though the number of HAPs is equal, the number of user antennas has been increased to provide more sub-channels (or diversity paths) of communication links between the transmitter and the receiver. The configuration (2,2) has 4 sub-channels, and (2,4) has 8 sub-channels. Therefore, (2,4) can provide a higher diversity reception gain. In addition, it can be observed from Figure 4 that the performance of (4,4) is better than that of (4,2), and the performance of (4,2) is better than that of (2,2), while the performance of (1,1) is the worst. Therefore, this further illustrates that multiple HAPs can indeed provide better system performance than a single HAP. As shown in Figure 5, the performance of (2,4) is better than that of (4,2), which is explained by the fact that multiple antennas at the receiver give a better performance improvement than that of multiple antennas at the transmitter, when the number of total antennas is equal.

Thirdly, Figures 6 and 7 depict the ASER versus the SNR with various HAP and user antenna configurations for BPSK and QPSK respectively. The Monte-Carlo simulation results validate the effectiveness of the analysis. As shown in Figures 6 and 7, the ASER decreases when the average SNR increases. We also observe that ASER is effectively decreased when the number of HAPs and user antennas increases. For example, when BPSK modulation is used, *γ* = 10 dB, and the network configurations are (4,4) and (2,2), the ASER performances are 16.8 dB and 3.5 dB better than that of a single antenna configuration, *i.e.*, (1,1). When QPSK modulation is used, *γ* = 15 dB, and the network configurations are (4,4) and (2,2), the ASER performances are 10.1 dB and 3.1 dB better than that of a single antenna configuration.

Finally, Figure 8 shows the ergodic capacity changes along with the SNR variation for different network configurations. From Figure 8, we can observe that the ergodic capacity, which is obtained by the perfect CSI and power allocation using the water-filling algorithm, is superior to that which is obtained by the unknown CSI and equal power allocation scheme, for the case of multiple HAP and user antenna network configurations. However, the ergodic capacity is the same for the case of single HAP and single user antenna configuration. As the SNR increases, the performance of the equal power allocation scheme gradually tends towards that of the water-filling scheme. Figure 9 shows the ergodic capacity changes along with the Rician factor *κ* under different SNR (*N_H_* **=** 4, *N_r_* = 4). We can discover that the ergodic capacity obtained by perfect CSI and power allocation using the water-filling algorithm is superior to that which is obtained using unknown CSI and equal power allocation scheme, for various SNR cases. Furthermore, we can see whether it is the perfect CSI or the unknown CSI, the Rician factor *κ* has the detrimental effect on the system capacity. If *γ* is −10 dB, **−**5 dB, 0 dB, 5 dB, and 10 dB, the *κ* has little effect on the system capacity when *κ* at small values. However, when *κ* is larger, e.g., *κ* = 20 dB, it seriously affects the system capacity. Additionally, as *γ* is increased, the extent of the damage increases. Therefore, we can observe that the LOS signal has a negative effect on the system capacity in HAPs network utilizing V-MIMO techniques.

## 5. Conclusions

This paper has investigated the diversity performance on multiple HAPs via V-MIMO transmission in the WSNs. Firstly, a virtual MIMO system model has been established by modeling a constellation utilizing interconnected HAPs, and the concept of MAV has been proposed. Secondly, assuming the channel is shadowed Rician fading, the PDF and CDF of received SNR have been derived. The ASER with BPSK and QPSK have then been given. When BPSK modulation is used, *γ* = 10 dB, and the network configurations are (4,4) and (2,2), the ASER performances are 16.8 dB and 3.5 dB better than that of (1,1). When QPSK modulation is used, *γ* = 15 dB, and the network configurations are (4,4) and (2,2), the ASER performances are 10.1 dB and 3.1 dB better than that of (1,1), respectively. Additionally, the system capacity has been investigated for a perfect CSI and an unknown CSI respectively. Most significantly, we discovered that the more visible HAPs can enhance the signal energy, and provide rate and integrity improvements. To best of our knowledge, this study has been the first time that the ergodic capacity with various SNR and Rician factors for different network configurations has been analyzed. We have found that the LOS signal damages the HAPs system capacity as the Rician factor is increased. Finally, the simulation has verified the effectiveness of our design and performance analysis. We conclude that the V-MIMO with multiple HAPs is a promising solution for future high-data-rate and frequency-efficient smart wireless sensor networks. In the paper, the MAV is 100% by default. In fact, the degree of overlapping coverage is always different, especially for multiple sensor devices in the overall networks. Therefore, further studies should extend the MAV to the possible percentage of overlapping coverage. In addition, we have mainly investigated the diversity performance provided by multiple HAPs. The multi-hop transmission (relay) can also increase the transmission performance of wireless systems. Therefore, one of the next steps to this work will be the research on multi-hop relay transmission in the HAPs networks.

## Figures and Tables

**Table 1:** Notations.

**Notation**	**Description**	**Notation**	**Description**
E [·]	The expectation operator	*s_ij_*	The RF signal voltage for the jth HAP
*N_H_*	The number of HAPs	*S_i_*	The signal power per transmitter antenna
*N_r_*	The number of receiving antennas	*α*^2^	The power of LOS component
*γ*	The SNR of receiver	*γ_th_*	The threshold of SNR for sensor device
‖·‖*_F_*	The Frobenius norm operator	*H_LOS_*	The channel matrix of shadowed LOS path
|·|	The determinant operator	*H_NLOS_*	The channel matrix of NLOS path
*tr*(·)	The trace operator	2*σ*^2^	The the power of NLOS component

**Table 2:** Parameters Used in Simulations and Their Values.

**Parameter**	**Value**	**Parameter**	**Value**
*N_H_*	1, 2, and 4	*κ*	1 dB
*N_r_*	1, 2, and 4	*γ*	10 dB
*a*	1, 2	*γ_th_*	10 dB
*b*	1, *sin*^2^(*π*/4)	*δ*	3.5 dB
